# Tissue-engineered and autologous pericardium in congenital heart surgery: comparative histopathological study of human vascular explants

**DOI:** 10.1093/ejcts/ezae027

**Published:** 2024-01-30

**Authors:** Katja Eildermann, Maksim Durashov, Kira Kuschnerus, Andrea Poppe, Viktoria Weixler, Joachim Photiadis, Matthias Sigler, Peter Murin

**Affiliations:** Department of Pediatric Cardiology and Intensive Care Medicine, Georg-August University Göttingen, Göttingen, Germany; Department of Pediatric Cardiology and Intensive Care Medicine, Georg-August University Göttingen, Göttingen, Germany; Department of Congenital Heart Surgery—Pediatric Heart Surgery, Deutsches Herzzentrum der Charité, Berlin, Germany; Department of Pediatric Cardiology and Intensive Care Medicine, Georg-August University Göttingen, Göttingen, Germany; Department of Congenital Heart Surgery—Pediatric Heart Surgery, Deutsches Herzzentrum der Charité, Berlin, Germany; Department of Congenital Heart Surgery—Pediatric Heart Surgery, Deutsches Herzzentrum der Charité, Berlin, Germany; Department of Pediatric Cardiology and Intensive Care Medicine, Georg-August University Göttingen, Göttingen, Germany; Department of Congenital Heart Surgery—Pediatric Heart Surgery, Deutsches Herzzentrum der Charité, Berlin, Germany

**Keywords:** Congenital heart disease, biocompatibility, histology, tissue-engineering, patches material, pericardium

## Abstract

**OBJECTIVES:**

The goal of this histological study was to assess the biocompatibility of vascular patches used in the repair of congenital heart defects.

**METHODS:**

We examined tissue-engineered bovine (n = 7) and equine (n = 7) patches and autologous human pericardium (n = 7), all explanted due to functional issues or follow-up procedures. Techniques like Movat-Verhoeff, von Kossa and immunohistochemical staining were used to analyse tissue composition, detect calcifications and identify immune cells. A semi-quantitative scoring system was implemented to evaluate the biocompatibility aspects, thrombus formation, extent of pannus, inflammation of pannus, cellular response to patch material, patch degradation, calcification and neoadventitial inflammation.

**RESULTS:**

We observed distinct material degradation patterns among types of patches. Bovine patches showed collagen disintegration and exudate accumulation, whereas equine patches displayed edematous swelling and material dissolution. Biocompatibility scores were lower in terms of cellular response, degradation and overall score for human autologous pericardial patches compared to tissue-engineered types. The extent of pannus formation was not influenced by the type of patch. Bovine patches had notable calcifications causing tissue hardening, and foreign body giant cells were more frequently seen in equine patches. Plasma cells were frequently detected in the neointimal tissue of engineered patches.

**CONCLUSIONS:**

Our results confirm the superior biocompatibility of human autologous patches and highlight discernible variations in the changes of patch material and the cellular response to patch material between bovine and equine patches. Our approach implements the semi-quantitative scoring of various aspects of biocompatibility, facilitating a comparative quantitative analysis across all types of patches, despite their inherent differences.

**Figure:**
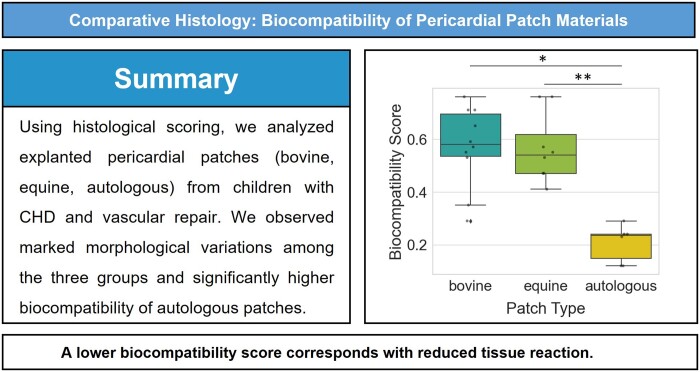

## INTRODUCTION

The use of patch material is inevitable in many surgical procedures for the correction or the palliation of congenital heart disease (CHD). Glutaraldehyde-treated autologous pericardium is preferentially used because of its ready availability and its high calcification resistance compared to other patch materials [1]. Concern has been raised over collagen cross-linking induced by glutaraldehyde (GA) in children due to its potential cytotoxicity [2]. In adults, the use of autologous and xenogeneic pericardium has shown satisfactory results in the repair of acquired heart disease with limitations in long-term performance due to calcification [3]. In redo operations, due to the lack of autologous tissue, several xenogeneic alternatives have been used with variable results [4–7]. Decellularization has been proposed to improve the immune-compatibility of the tissue and at the same time to preserve its structure and biomechanical properties [8–10]. Our group has recently published encouraging early clinical results of Matrix Patch (Auto Tissue Berlin GmbH, Berlin, Germany), a tissue-engineered, cell-free, equine-derived pericardium processed without the use of GA to delay calcification [11, 12]. Several other alternatives were reported in the literature based on clinically relevant outcome measures such as reinterventions or loss of function due to calcification and endocarditis [5, 13]. Although these variables were shown to be relevant for the clinical outcome, real-world histopathological data on long-term integration and rejection of the different materials are largely missing [8, 14].

The goal of this histopathological study was to identify qualitative differences among 3 materials: Matrix Patch, CardioCel Patch and human autologous pericardium. It also sought to implement a semi-quantitative scoring system for various aspects of biocompatibility, facilitating a comparative analysis across all patch types.

## MATERIALS AND METHODS

### Patch materials

We examined the following 3 patch materials:

- The CardioCel Patch (LeMaitre, Burlington, MA, USA) is crafted from decellularized and GA–cross-linked bovine pericardial tissue and has undergone the company's proprietary ADAPT treatment to prevent calcification [15].
- The Matrix Patch (Auto Tissue Berlin GmbH, Berlin, Germany) is made from decellularized equine pericardium without the use of GA.
- Human pericardial patches are crafted from autologous pericardium obtained during surgery. Excised pericardium was incubated in a 0.625% GA solution for 3–4 min and thoroughly rinsed to remove any remaining GA prior to being implanted.

Both tissue-engineered patches were used according to the manufacturers’ instructions.

### Ethical considerations

This single-centre, retrospective study was conducted with the approval of our institutional review board (EA2/081/20, approved on 4 June 2020). The need for informed consent for anonymized data analysis was waived. Both tissue-engineered patches used in this study are certified for use in vessel augmentation, heart valve reconstruction and the closure of intracardiac septal defects.

### Surgical considerations

In general, patch augmentation was used only when there was a significant reduction in the cross-sectional area of the narrowed segment and when neither augmentation with native tissue nor a simple incision was applicable. Whereas autologous pericardium was typically the material of choice for vascular reconstructions, tissue-engineered patches were used as an alternative when autologous pericardium was unavailable or deemed unsuitable by the surgeon. The choice of a specific tissue-engineered patch depended on the surgeon’s preference and the quality of the local tissue. Efforts were made to avoid tissue redundancy and to accurately recreate the 3-dimensional geometry of complex patches.

### Patch retrieval

The patch specimens used in this study were implanted between September 2013 and January 2022 and retrieved between August 2015 and August 2022 either due to loss of function (e.g. stenosis or aneurysm) of the vascular structure or during planned redo surgery at the patch implantation site (planned staged surgery). For this study, we focused on paediatric patients who received 1 of these materials for vascular reconstruction during repair of CHD.

### Study objectives

The goal of this study was to investigate biological reactions within and around implanted biological vascular patches. On the luminal side, we focused on thrombus and excessive pannus formation with abundant granulation tissue and immune cell infiltrations. The patch material was evaluated for degenerations, calcification, exudate, immune cell accumulation at the patch tissue interface and the presence of foreign body giant cells. Abluminal tissue was examined for neoadventitial inflammation, e.g. lymphoid tissue. We then wanted to determine patch-type specific histological patterns of tissue reaction to finally implement a scoring system of the extent and severity of the mentioned aspects of biocompatibility, facilitating a comparison across all patch types despite their inherent differences.

### Histopathological workup

Explants were fixed in 4% formalin, embedded in paraffin and processed as usual to obtain histological sections. Calcified patches or parts of the patches containing suture material were unsuitable for paraffin embedding and microtome sectioning and thus were embedded in the synthetic resin Technovit 9100 (Technovit, Heraeus-Kulzer, Hanau, Germany) as described previously [16]. After hardening, resin blocks were sectioned with a diamond saw (Exakt, Norderstedt, Germany), ground to approximately 50 µm thickness using a precision grinder (Exakt).

In addition to standard stainings and in order to gain a comprehensive understanding of the tissue components, Movat Pentachrom after Verhoeff (referred to as Movat Verhoeff in this paper) staining was applied to all specimens (#12061, Morphisto GmbH, Offenbach, Germany). This five-colour stain visualizes nuclei in blue-black, muscle and cytoplasm in red, ground substance in blue/turquoise, collagen in yellow, cartilage in blue-green/yellowish, elastic fibres in black, bone in yellow/red and fibrin in bright red. Immunohistochemical (IHC) analyses were conducted according to standard protocols. Details on antigen retrieval, primary and secondary antibodies and their dilutions are provided in Table 1. The visualization reaction utilized 3,3'-diaminobenzidine (DAB), which resulted in a brown coloration. Negative control staining used immunoglobulins instead of the primary antibody in respective concentrations. Slides with IHC stainings were counterstained with haematoxylin (Sigma Aldrich, St Louis, MO, USA). All histological slides were digitized using the dotSlide system (Olympus, Tokyo, Japan) and analysed with the cellSens Dimension software (Olympus).

**Table 1:** Imunohistochemical specifications

Primary antibodies	Dilution	Target retrieval	Secondary antibodies	Target
CD15	1:1.000	High PH	Polymer	Neutrophils
CD163	1:500	High PH	Polymer	M2 Macrophages
CD68	1:200	High PH	Polymer	Macrophages
CD3	1:500	pH6.1	Polymer	T-Lymphocytes
CD79a	1:100	pH6.1	Polymer	B-Lymphocytes
SMA	1:300	Citrat	Anti-mouse	Contracting cells
Vimentin	1:500	Citrat	Anti-mouse	Somatic cells
CD138	1:200	High PH	Polymer	Plasma cells
CD31	1:100	High PH	Polymer	Endothelial cells
CD45	1:3000	pH6.1	Polymer	Leucocytes
Fibrin	1:500	Citrat	Anti-mouse	Fibrin
CD42b	1:600	High PH	Polymer	Platelets

Primary antibodies: mouse anti-human CD15 (Zytomed, Berlin, Germany) (MSK108-05); mouse anti-human CD163 (BioRad, Hercules, CA, USA) (MCA1853); mouse anti-human CD68 (Dako, Glostrun, Denmark) (M0876); polyclonal rabbit anti-human CD3 (Dako, A0452); mouse anti-human CD79a (Dako, M7050); mouse anti-human smooth muscle actin (SMA) (Dako, M0851); mouse anti-human CD138 clone MI15 (Dako, M0751); mouse anti-human CD31 (Dako, M0823); mouse anti-human CD45 (Cell Marque, 145M-96); mouse anti-human fibrin ß-chain (Biomedica Diagnostics (Windsor, ON, Canada), ref 350); and rabbit anti-human CD42b (Abcam, PLC, Cambridge, United Kingdom, ab227669). Target retrieval: High pH: EnVision Flex Target Retrieval Solution high pH, (Dako, K8004); pH6.1: target retrieval solution pH6,1 (Dako, S1699); Citrat: Target Retrieval Solution Citrat pH 6 (Dako, S2369). Secondary antibodies: polymer: Zytochem plus horseradish peroxidase/polymer system mouse/rabbit (Zytomed, POLHRP-100); anti-mouse: polyclonal rabbit anti-mouse immunoglobulins/horseradish peroxidase (Dako, P0260).

### Evaluation

To compare tissue reactions to different biological patch materials, we assessed, scored and documented a variety of biocompatibility features. Unless otherwise stated, a score of 0 was given when a feature was absent, 1 when a feature was present and/or above normal levels and 2 when there was a significant manifestation of that feature.

Histochemical and immunohistological stainings were evaluated, and the scores were recorded. To consolidate the data, features that were related to the same aspect were grouped, using the highest score from any individual feature score within that group. This method resulted in a set of 7 aspects of biocompatibility that are described in detail below.

**Thrombus** was evaluated considering Movat Verhoeff, fibrin and CD42b stains. A thrombus coverage of ≥ 75% of the surface received a score of 2, whereas partial thrombus coverage received a score of 1 and the absence of thrombus earned a score of 0.

The **extent of luminal neotissue formation** was quantified by measuring its width in the area of its greatest extent using ImageJ (National Institutes of Health. Version 1.54f, https://imagej.nih.gov/ij/). A thickness of < 100 µm was scored 0; measurements between 100 µm and 700 µm were scored 1; and > 700 µm scored 2. A limit of 700 µm was chosen because this thickness usually exceeded that of the patch itself.

Importantly, in this study, the aspects ‘thrombus’ and ‘extent of luminal neointimal formation’ collectively represented endothelialization. The presence of endothelium can be presumed when neotissue has formed on the luminal surface of the patch, whereas the absence of or a state of damaged, activated or incomplete endothelium can be inferred from the presence of thrombus or platelets on the surface. Areas without thrombus and without neotissue were usually endothelialized in our specimen, as determined by morphological analysis.

**Pannus inflammation,** such as the presence of granulation tissue within the luminal tissue, was evaluated employing Movat Verhoeff staining, considering CD31 for vascularization, CD45 for leucocytes, CD138 for plasma cells and CD15 for neutrophils. A score of 0 was given for no significant findings, 1 when inflammation was notable and 2 when there were significant immune cell accumulations in larger areas.

The **cellular response to the patch material** involved multiple features, including cellular accumulation at both the luminal and abluminal patch–tissue interfaces and the presence of foreign body giant cells. Scores were assigned and consolidated as described above by evaluating macrophages (CD163, CD68), somatic cells (vimentin) and leucocytes (CD45).

The aspect of **patch degradation** combined the assessment of visible dissolution and/or dislocation of the patch material itself and the presence of exudate. It was evaluated with CD15, CD68 and Movat Verhoeff stains.

**Calcifications** were identified by von Kossa staining and scored according to its contribution to tissue hardening.

**Neoadventitial inflammation** was scored on the presence of leucocytes (CD45) in general and more specifically by the presence of plasma cells (CD138) in neoadventitial tissue. Lymphoid tissue was considered the most relevant sign for neoadventitial inflammation and scored as a 3.

Scores were noted and consolidated as described (Supplemental Table 1). The average of all 7 aspect scores resulted in a single biocompatibility score for each specimen. For a comparative analysis of bovine, equine and human samples, scores for thrombus formation, pannus thickness, inflammation, cellular response, patch degradation, calcification, neoadventitial inflammation and the overall score were summarized per group, and mean values were visualized as a heat map. Summary statistics were visualized as box and whiskers blots (Supplemental Fig. 1). Statistical data analysis and data visualizations were performed using Python 3-based libraries including Pandas, pearsonr, matplotlib, Seaborn and NumPy. Applied statistical tests are detailed in the respective results sections.

## RESULTS

A table of all of the results is provided in the Supplemental Table.

### Study population

**Figure 1:**
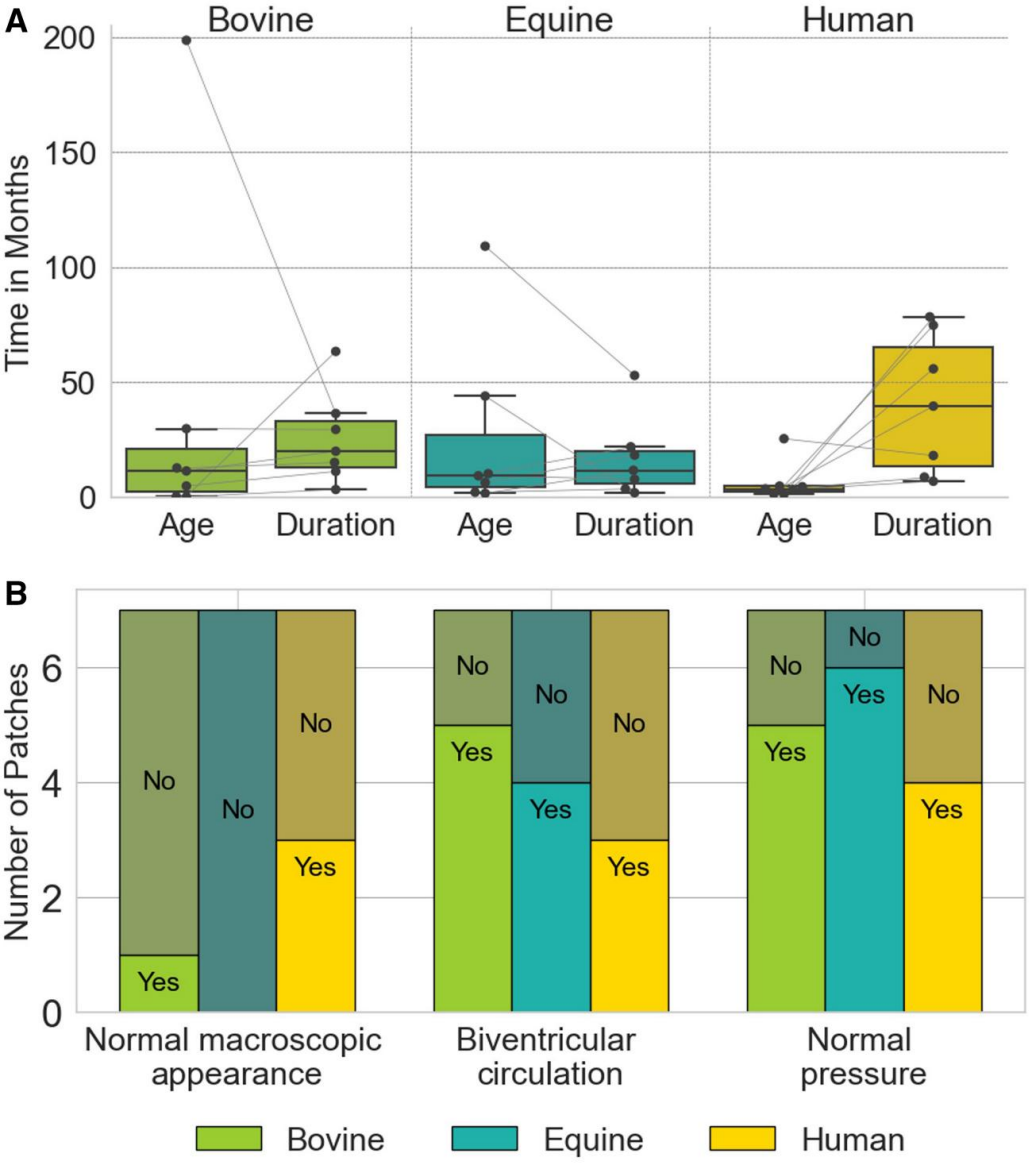
Characteristics of the study population in bovine, equine and human pericardial patches. (**A**) Age at implantation and duration of implantation. Box plots represent data distribution with individual points shown by swarm plots. (**B**) Distribution of clinical observations. Bars represent counts for each observation, labelled as ‘Yes’ or ‘No’ based on the presence or absence of the specific clinical feature.

The data set utilized in this study included 21 vascular patches distributed across 3 groups: bovine pericardium (*n* = 7), equine pericardium (*n* = 7) and human pericardium (*n* = 7). Summarized patient characteristics comparing these 3 groups are provided in Table 2 and Fig. 1A and B. Patients who received an equine patch were, on average, younger and had a longer mean duration of an implant, as shown in Fig. 1A. However, neither ‘age at implantation’ (*P*-value = 0.3578, determined by the Kruskal-Wallis test for not normally distributed data) nor ‘duration of implantation’ (*P* = 0.1900, as determined using an analysis of variance test for normally distributed data, where normal distribution was assessed using the Shapiro-Wilk test) were significantly different.

**Table 2:** Summary of grouped patient information

	Bovine	Equine	Human
Total number of specimens	7	7	7
Site of explantation: PA	4	7	7
Site of explantation: AA	2	0	0
Site of explantation: VC	1	0	0
Duration of implantation median (IQR) in months	19.97	9.59	11.7
	(13.16–32.9)	(13.42–65.28)	(5.7–20.09)
Age at time of implantation median (IQR) in months	11.4	3.61	9.3
	(2.6–21.24)	(2.28–4.68)	(4.27–27.09)
Reason for explantation: Stenosis	5	6	3
Reason for explantation: Planned surgery on implant site	1	0	2
Reason for explantation: Aneurysm	1	1	2
Abnormal macroscopic appearance	6	7	4
Biventricular circulation	5	4	3
Systemic pressure at patch site	2	1	3

AA: aortic arch; IQR: interquartile range; PA: pulmonary artery; VC: vena cava.

The primary reason for explantation of both bovine and equine patches was stenosis, with lower incidences of aneurysm (*n* = 4) and planned surgery (*n* = 3). Only bovine patches included samples from non-pulmonary artery (PA) locations, with 2 from the aortic arch and 1 from the vena cava, whereas all equine and human specimens were explanted from the PA. Figure 1B illustrates the distribution of the categorical (Yes/No) variables: normal macroscopic appearance (*P* = 0.12), biventricular circulation (*P* = 0.56) and normal systemic pressure at the patch site (*P* = 0.50) in each group. The χ^2^ test of independence, commonly used to assess whether there is a significant association between 2 categorical variables, resulted in non-significant statistical outcomes for all 3 parameters.

### Histopathological assessment

**Figure 2:**
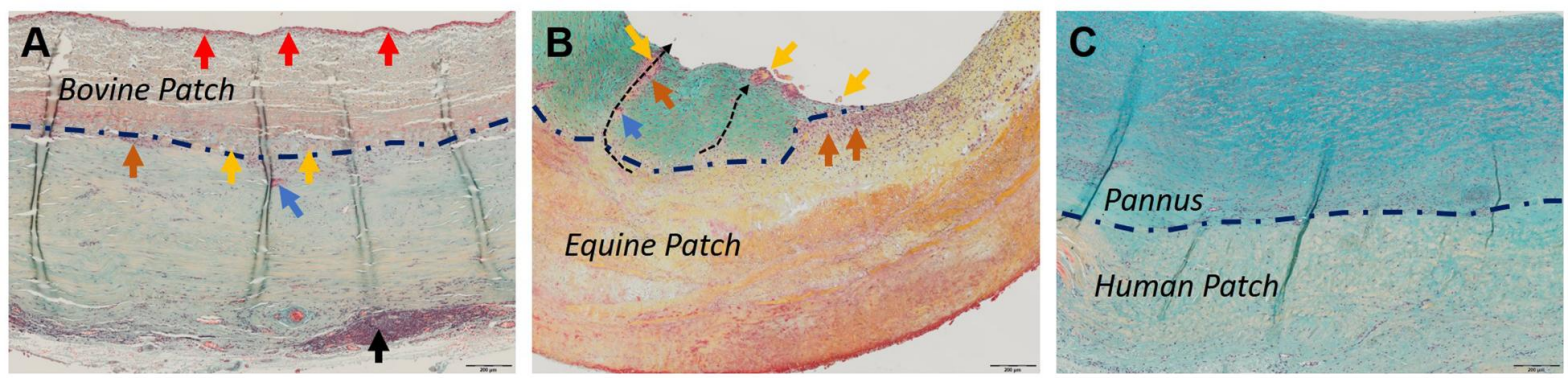
Microscopic images of selected luminal surfaces **(**Movat-Verhoeff stain). (A) Bovine patch with thrombus (red arrows). Black arrows point to lymphoid tissue. (**B**) Equine patch with pannus (turquoise staining) and patch fragments (yellow arrows) being transported towards the luminal surface (black dotted arrows) by macrophages (orange arrows)/foreign body giant cells (blue arrows). (**C**) Human autologous patch with non-inflammatory pannus. Blue dashed line indicates the patch-tissue borders in 2A–C.

Thrombus and pannus thicknesses were determined (Fig. 2A–C). Whereas equine and human patches did not show thrombus adhesions, 2 bovine patches had > 75% of their surface covered with thrombus, and 1 bovine patch showed partial thrombus adherence (Fig. 2A). Tissue thickness below 700 µm without thrombus apposition was considered unremarkable concerning biocompatibility. This criterion was met in 1 out of 7 bovine patches, in 3 out of 6 equine patches and in 4 out of 7 autologous patches (Fig. 2C). Correspondingly, although all human and equine patches exhibited complete endothelialization in the analysed regions, endothelialization was incomplete in 3 out of 7 bovine patches, as determined morphologically.

### Pannus inflammation

Luminal neotissue was assessed for the presence of inflammatory features such as granulation tissue formation, the presence of plasma cells (CD138) and/or neutrophils (CD15). Although these immune cells were present in a majority of bovine (6 of 7) and in all equine patches that allowed for this analysis, only a minority of human pericardial patches (2 of 8) showed these signs of inflammation.

#### Histological content of patch materials

**Figure 3:**
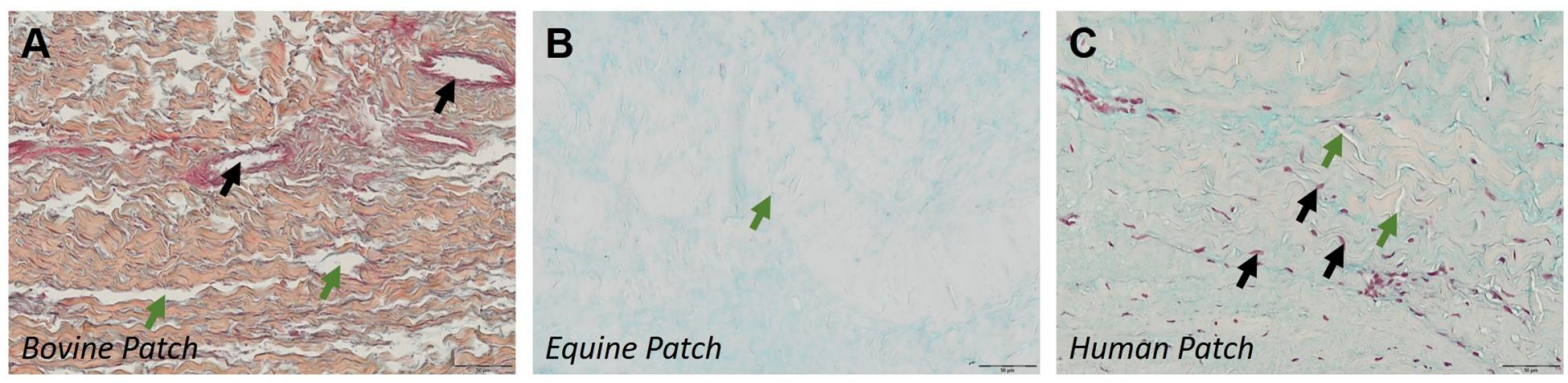
Appearance of pericardial patch materials. (**A**) Bovine patch; Collagen stained orange. Black arrows: remnant vessels. Green arrows: inter-collagenous spaces. (**B**) Equine patch with small gaps (green arrow) between collagen fibres (pale orange stain). (**C**) Autologous human patch. Black arrows point to nuclei; green arrows, to collagen gaps.

Bovine patch material was characterized by compact collagen fibres with distinct edges, interspersed with noticeable gaps (Fig. 3A). Equine (Fig. 3B) and human (Fig. 3C) patches showed wavy, densely packed, less discernible collagen fibres with minimal gaps between them. Examining the tissue for residual vascular structures and residual cells revealed distinct features for the 3 groups. Bovine samples displayed remnants of original vessels (Fig. 2A) and associated cellular structures, but in the absence of detectable interstitial cells. Equine samples, devoid of any residual cell material, showed former vessel regions solely through localized variations in the extracellular matrix. Conversely, human patches contained interstitial cells (Fig. 3C) and vessels, resembling the morphology of typical pericardial tissue.

When measured upon explantation, bovine patches showed a mean diameter of 535 µm with an interquartile range (IQR) of 475–584 µm and equine patches, a mean diameter 699 µm with a large IQR of 528–972 µm, respectively, which was attributed for the most part to edematous infiltrations in some of the patches. For autologous tissue, differentiation of patch and abluminal tissue was challenging due to the many morphological similarities. In the latter, the average diameter was 680 µm with an IQR of 480–780 µm. However, this result should be looked at with caution due to the mentioned uncertainties.

#### Cellular response and patch degradation

Bovine patch material showed few cell infiltrations. Newly formed extracellular matrix, fibroblasts and macrophages were incidentally found in spaces between collagen fibres, mainly in peripheral regions. In contrast, equine patches did not exhibit such colonization into the matrix. Quantifying cellular infiltration in autologous patches was challenging due to the presence of pre-existing cellular components within the biomaterial. Notably, the presence of blood vessels and signs of extravasation indicated the potential for active cellular recolonization. This feature was not rated in our biocompatibility score.

**Figure 4:**
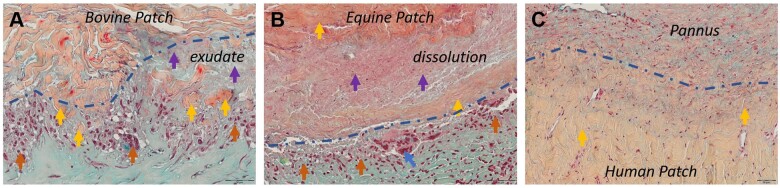
Degradation patterns of patch materials. Movat Verhoeff stained patch materials. (**A**) Bovine patch. Collagen fragments (yellow arrows), exudate (lilac arrow), macrophage accumulation at patch–tissue interface (blue dashed line, orange arrow). (**B**) Equine patch. Dissolution and edematous swelling of patch material (yellow, lilac arrows), macrophages (orange arrow) and foreign body giant cells (blue arrows) at patch–tissue interface (blue dashed line). (**C**) Human patch—No cell accumulation, no discernible degradation pattern at patch–tissue interface (blue dashed line).

The macrophage accumulations, which ranged from a loose collection of a few cells to extensive clustering of polymorphonuclear cells, and the formation of a dense macrophage wall with vertically aligned macrophages were frequently observed and were locally related to the patch–tissue interface (Fig. 4A–C). This reaction was more pronounced on the abluminal side, and equine patches were slightly more affected than bovine patches. No such cell accumulations were observed around autologous patch parts (Fig. 4C).

In this context, foreign body giant cells deserve special attention as they are instrumental in the degradation of foreign materials. Interestingly, these cells were present only on the abluminal side in most bovine patches and were found on either side in all equine patches, predominantly within macrophage accumulations in the patch–tissue interface, but notably also within the neotissue and protruding towards the lumen/abluminal space (Fig. 2B).

In bovine patches, degradation was marked by visible fibre disintegration, chiefly in the abluminal periphery (Fig. 4A). This situation often coincided with the presence of exudate, an amorphous, acellular substance rich in small particles that showed a dotted positive reaction in CD68 and CD15 IHC.

In about half of the equine patches, degradation was distinct and characterized by dissolution rather than disintegration (Fig. 4B). Dissolution was accompanied by edematous swelling that caused an expansion of the patch diameter. Visible collagen fragments were seen only rarely. Notably, 1 equine specimen exhibited very pronounced immune cell infiltration and significant patch degradation. In autologous patches, no distinct degradation pattern or evident exudate was observed in contrast to both bovine and equine patches (Fig. 4C).

### Calcification

**Figure 5:**
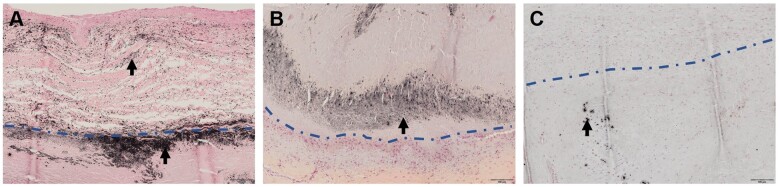
Von Kossa staining highlights calcification. (**A)** Bovine patch with calcification accumulation (black arrows) at abluminal patch–tissue interface (blue dashed line) protruding into the patch. (**B**) Equine patch with calcified particles (black arrows) observed within edematously swollen area. (**C**) Human autologous patch with presence of calcifications within the patch material.

Bovine patches exhibited a distinctive, speckled pattern of microcalcification, overlapping with exudate accumulation at the abluminal patch–tissue interface and extending into the abluminal side of the patch (Fig. 5A). Of the 7 bovine patches examined, 3 were significantly hardened, whereas 1 showed mild hardening. In equine patches, calcification also overlapped with the presence of exudate (Fig. 5B). However, in contrast to bovine patches, where the primary calcification deposits were located at the patch–tissue interface, calcifications in equine patches were found within the patch material itself, presenting as a nanoscale veil-like pattern with varying densities. Autologous tissues displayed sporadic macro- and microcalcifications that were not associated with exudate or patch degradation. For example, 1 autologous patch had significant macrocalcifications in the abluminal tissue, extending into the patch itself. Another exhibited calcification in a cartilage-like area near surgical sutures (Fig. 5C), without any direct local relation.

### Abluminal tissue

The abluminal tissue generally exhibited high vascularization and high collagen content. Immune cell and notably plasma cell infiltrations were commonplace in bovine and equine patches, but less prevalent in those of human origin. Lymphoid tissue formation was detected in the majority of bovine patches (Fig. 3A, black arrow), in some equine patches and in only 1 human patch. Compared to bovine and equine materials, autologous tissue typically exhibited a lower extent of abluminal tissue formation.

### Comparing biocompatibility

**Figure 6:**
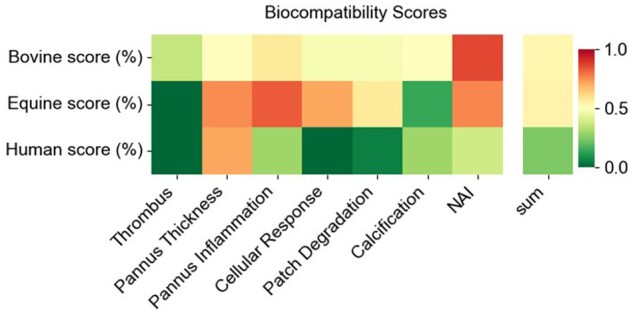
Biocompatibility scores. Heat maps showing the summarized aspect scores for bovine, equine and autologous patch materials. The lower the score indicates better biocompatibility. NAI: neoadventitial inflammation.

Biocompatibility scores were displayed as heat maps showcasing the averages of each aspect for each group (Fig. 6), and as box-and-whisker plots with data points and the average, highlighted in red (Supplemental Fig. 1). To evaluate statistically significant differences in the expression of each aspect between the groups, the non-parametric Mann–Whitney U test was employed. The analysis revealed no significant differences (*P* > 0.05) between the groups for ‘thrombus’ formation, ‘extent of luminal tissue’, ‘pannus inflammation’, ‘calcification’ and ‘neoadventitial inflammation’. However, notable exceptions were observed for the aspects of ‘cellular response to patch material’ (bovine vs human, *P* = 0.0097; equine vs human, *P* = 0.0027), ‘patch degradation’ (bovine vs human, *P* = 0.031; equine vs human, *P*-value = 0.008) and the overall ‘biocompatibility score’ (bovine vs human, *P* = 0.014; equine vs human *P* = 0.0062), which showed statistically significant differences between both tissue-engineered and the human autologous pericardial patches.

The correlation between each aspect and the duration of implantation were assessed in a Spearman's correlation analysis. No correlation was found for the aspects ‘thrombus’ (ρ = −0.025, *P* = 0.91), ‘extent of luminal tissue’ (ρ = −0.160, *P* = 0.499), ‘patch degradation’ (ρ = −0.186, *P* = 0.418), ‘neoadventitial inflammation’ (ρ = −0.093, *P* = 0.687) and the overall biocompatibility score (ρ = −0.29, *P* = 0.197). However, ‘pannus inflammation’ (ρ = −0.476, *P* = 0.029) showed a significant negative correlation and the aspect ‘cellular response to patch material’ (ρ = −0.429, *P* = 0.052) was close to statistical significance. Interestingly, calcification was the only aspect with a tendency for a positive correlation (ρ  =  0.304, *P*-value = 0.180), although it was not significant.

We also tested for statistically significant differences in biocompatibility scores with respect to macroscopic dysfunction, biventricular circulation, systemic pressure and the various reasons for explantation. Respective box and whiskers blots are presented in Supplemental Fig. 2. No significant findings were observed.

## DISCUSSION

Surgical repair of CHD often requires the use of patch materials. Due to the limitations of synthetic materials, there has been a shift towards using biological tissues, particularly pericardium, from either the same individual (autologous) or animals (xenograft) [3]. Articles in the literature offering direct comparisons between biological patch materials are sparse, and surgeons often have to base their choice on personal preference rather than on empirical evidence. However, a study comparing autologous pericardium with tissue-engineered patches highlighted its lower reintervention rate in PA reconstructions, though clinical outcomes were comparable across tissue-engineered materials [17].

Addressing the gap of a missing comparative analysis of biological patch materials, our study investigated the differences among 3 materials commonly used in vascular repair: the Matrix Patch, the CardioCel Patch and the human autologous pericardial patch. The selection of these types of patches was based on the availability of more than 3 specimens of each type, enabling a thorough analysis of material characteristics upon implantation. We utilized all available specimens, which amounted to 7 per implant type.

In bovine patches, the larger gaps between the GA–cross-linked collagen fibres allowed for cellular infiltration at the periphery of the patch. Despite causing fibre dislocation and fragmentation, this infiltration did not dissolve the collagen scaffold itself. Our hypothesis is that degradative enzymes are ineffective in breaking down the GA–cross-linked collagen scaffold. However, small yet visible fragments of the scaffold, often surrounded by foreign body giant cells, were observed in the abluminal tissue, indicating active degradation.

The structurally unchanged equine patch was typically devoid of cells. Nonetheless, exudates and/or tissue fluids were observed permeating the material, resulting in edematous swelling. We identified small patch fragments that appeared to be structurally changed and were surrounded by macrophages and foreign body giant cells, migrating through the adjacent tissues towards the lumen and the abluminal spaces. In light of these observations, we postulated that swelling induces dissolution of patch material, facilitating cell–material interactions. We suggested that endogenous enzymes digest swollen equine material, leading to degradation. However, in our cohort, there was no obvious loss or replacement of material, possibly masked by swelling or tissue replacement. The critical issue is whether degraded material is effectively replaced by native tissue or leaves a gap. So far, no gaps in the vascular positions have been observed, and distinguishing native material and tissue replacement is difficult. Based on our current results, we cannot sufficiently determine whether the potential disappearance of patch material and of concomitant tissue replacement or the lack of such tissue replacement is likely to influence clinical outcomes. The answer could be particularly relevant in situations in which tissue replacement is exposed to greater mechanical and dynamic challenges, such as in valvular leaflets. The dissolution and degradation of patch material certainly merit detailed exploration in subsequent research.

Even though human autologous tissue was also fixed in GA, albeit for shorter incubation periods, neither obvious implant degradation nor pronounced cell–material interactions were observed. Although pannus formation was clearly distinguishable, differentiating newly formed abluminal tissue from the patch material proved challenging due to their similar appearances.

Whereas these qualitative differences represent inherent characteristics of the respective materials, the intensity of the expression of individual aspects of biocompatibility may depend on additional variables. These include the site of the implant with the related blood pressures, the patient's age at the time of the implant, the duration of the implant and the underlying congenital heart defect. With our low sample count, it was not feasible to construct groups with an even distribution of all potential confounders. The ensuing discussion on statistical outcomes is thus descriptive for our study cohort.

The implementation of a semi-quantitative scoring system facilitated the comparison of expression intensities for different aspects of biocompatibility. This system was designed to incorporate patch type-specific measures, particularly for aspects that exhibit inherent differences between patch types.

Although no significant differences were found between the 2 tissue-engineered materials, the aspects ‘cellular response to patch material’, ‘patch degradation’ and the overall ‘biocompatibility score’ were significantly lower in human autologous pericardial patches compared to both tissue-engineered types. This result aligns well with clinical experiences in which autologous material also demonstrates superior performance.

It is noteworthy that the extent of pannus formation was similar across the compared patch types. This observation suggests that it may be more heavily influenced by established factors, such as altered blood flow conditions, rather than by the patch material itself.

We also investigated whether the expression intensities of any aspects correlated with the duration of the implantation. Our findings revealed that shorter durations were associated with greater pannus inflammation and more intense cellular responses to the patch material. This result suggests that these aspects might represent a prolonged acute response that slowly resolves over time. Only calcification potentially increases with prolonged implantation times, although this observation was not statistically significant.

Despite numerous attempts to prevent the activation of the adaptive immune system, we observed a significant presence of plasma cells, especially pronounced in the abluminal region of bovine and equine patches.

Calcifications in tissue-engineered patches were often associated with exudate-rich regions. This exudate typically builds up as a result of ongoing chronic inflammation and necrosis. Dying cells, notably macrophages and neutrophils identified by CD15 and CD68 remnants in the exudate, release intracellular contents, including phosphates, which then combine with calcium ions in the tissue to form calcium phosphate crystals. The inability of dead tissue, including the patch material, to regulate calcium, may contribute to these calcium accumulations [18]. In addition, macrophages and other inflammatory cells, which are prevalent at the patch–tissue interface, secrete molecules that encourage calcium deposition [19]. These microcrystals gradually clump together, forming visible calcium deposits in the tissue that are inert and can persist indefinitely. Although equine patches exhibit high overall incidences of microcalcification similar to those of bovine patches, the tissues appeared less hardened, possibly due to less aggregation, as suggested by the veil-like distribution of the microcalcifications.

## LIMITATIONS

The quantitative outcomes are descriptive and are limited by the small sample size and the diversity of our patient cohort. Variability in potential confounders, both institutional and patient-specific, could introduce bias, because these variables were not evenly distributed across groups. The high variability in patients and the selective nature of the choice of patch type by the surgeons, especially in a single-institution study, are additional confounders.

Another significant limitation is the inherent bias in analysing patches from unscheduled surgical interventions, which are typically removed due to dysfunction. This factor skews the sample towards malfunctioning patches and limits the examination of those functioning effectively. To gain deeper insights into overall patch performance, our findings should be integrated with clinical outcomes.

## CONCLUSION

Our results indicate the superior biocompatibility of human autologous patches compared to animal-derived tissue-engineered patches, highlighting discernible variations in responses among bovine and equine patches. Furthermore, we have introduced a scoring system that facilitates the quantification of various aspects of material rejection accommodating their diverse qualities. The search for an ideal patch material for repair of congenital heart defects remains an ongoing challenge.

## Supplementary Material

ezae027_Supplementary_Data

## Data Availability

Raw data were generated at both sites. Derived data supporting the findings of the study are available from the corresponding author on request.

## Notes

A portion of the data were presented at the Annual Meeting of the German Society for Thoracic and Cardiovascular Surgery, Hamburg, Germany, 19–22 February 2022.

## ABBREVIATIONS

CHD: congenital heart disease
GA: glutaraldehyde
IHC: immunohistochemistry
IQR: interquartile range
NAI: neoadventital inflammation
PA: pulmonary artery
