# Supplementary material for: Tissue-engineered and autologous pericardium in congenital heart surgery: comparative histopathological study of human vascular explants
Source: Eur J Cardiothorac Surg. 2024 Jan 30;65(3):ezae027. doi: 10.1093/ejcts/ezae027 (PMC10924714; doi:10.1093/ejcts/ezae027)
Supplement: ezae027_Supplementary_Data [file ezae027_supplementary_data.zip › Supplemental Table.docx]

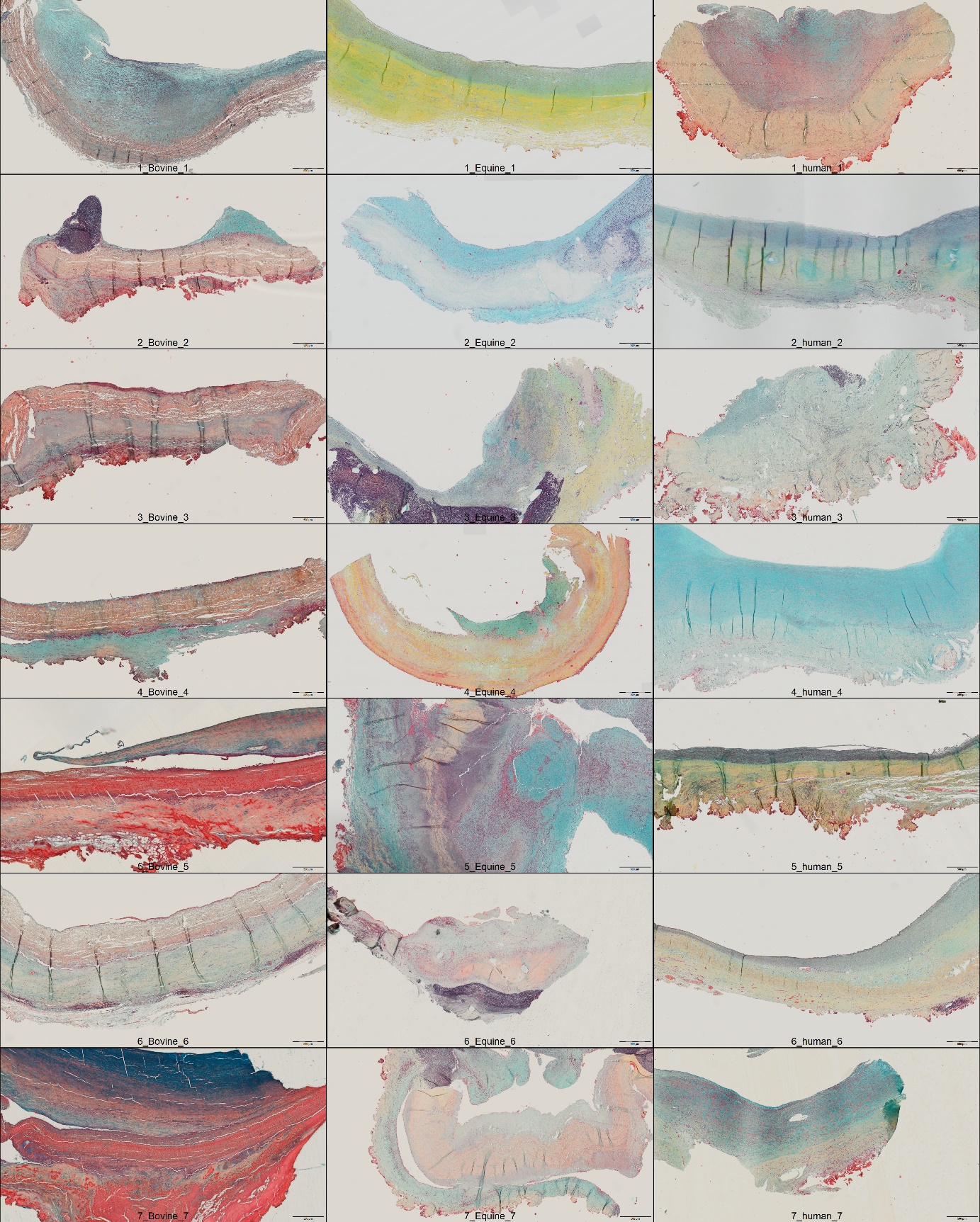


Figure 1: Movat Verhoeff stained overviews of all specimens used in this study. Sample ID within images.

Table 1: Raw Data for Scoring.

**Legend**

Reason for Explantation stenosis, planned (surgery at implant site), aneurysm

ExplantationSite PA, AA, VC

Age at Implantation in months

Duration of Implantation in months

Macroscopic_Dysfunction 0: No; 1: Yes

Biventricular_Circulation 0: Yes; 1: No

Blood_Pressure 0: normal; 1: abnormal

Thrombus 0: No; 1: <75% of surface; 2: complete surface

Extent of Luminal Tissue meassured at greatest expression; in µm; 0: <100µm; 1: 100µm < 700µm, 2: >700µm

Pannus Inflammation 0: Not detected; 1: present; 2: marked

Cellular response to Patch Material 0: Not detected; 1: present; 2: marked macrophage wall; 3: lymphoid tissue

Patch Degradation 0: Not detected; 1: present; 2: marked; 3: very pronounced

Calcification 0: Not detected; 1: present but low effect on tissue hardening; 2: tissue notably hardened

NeoAdventitalInflammation 0: Not detected; 1: present; 2: marked, e.g. plasma cells present; 3: lmphoid tissue

Biocompatibility Score Calculated as mean from the 7 aspects in frame

P_FBGC_luminal 0: not detected; 1: detected

P_FBGC_abluminal 0: not detected; 1: detected

width Luminal Tissue in µm

width Patch in µm

| ID | Bovine Scores | Equine Scores | Human Scores |
| --- | --- | --- | --- |
| 1 | 0,29 | 0,41 | 0,18 |
| 2 | 0,35 | 0,53 | 0,18 |
| 3 | 0,65 | 0,47 | 0,29 |
| 4 | 0,53 | 0,47 | 0,24 |
| 5 | 0,71 | 0,76 | 0,12 |
| 6 | 0,59 | 0,55 | 0,24 |
| 7 | 0,71 | 0,76 | 0,35 |
|  |  |  |  |
|  | 0,55 | 0,57 | 0,23 |

Table 2: Biocompatibility Score for each Explant.

|  | FBGC_Luminal | FBGC_Abluminal |
| --- | --- | --- |
| Bovine score (%) | 0,00 | 0,71 |
| Equine score (%) | 1,00 | 1,00 |
| Human score (%) | 0,00 | 0,00 |

Table 3: Foreign Body Giant Cells luminal and abluminal
