# Supplementary figures and images for: Tissue-engineered and autologous pericardium in congenital heart surgery: comparative histopathological study of human vascular explants
Source: Eur J Cardiothorac Surg. 2024 Jan 30;65(3):ezae027. doi: 10.1093/ejcts/ezae027 (PMC10924714; doi:10.1093/ejcts/ezae027)

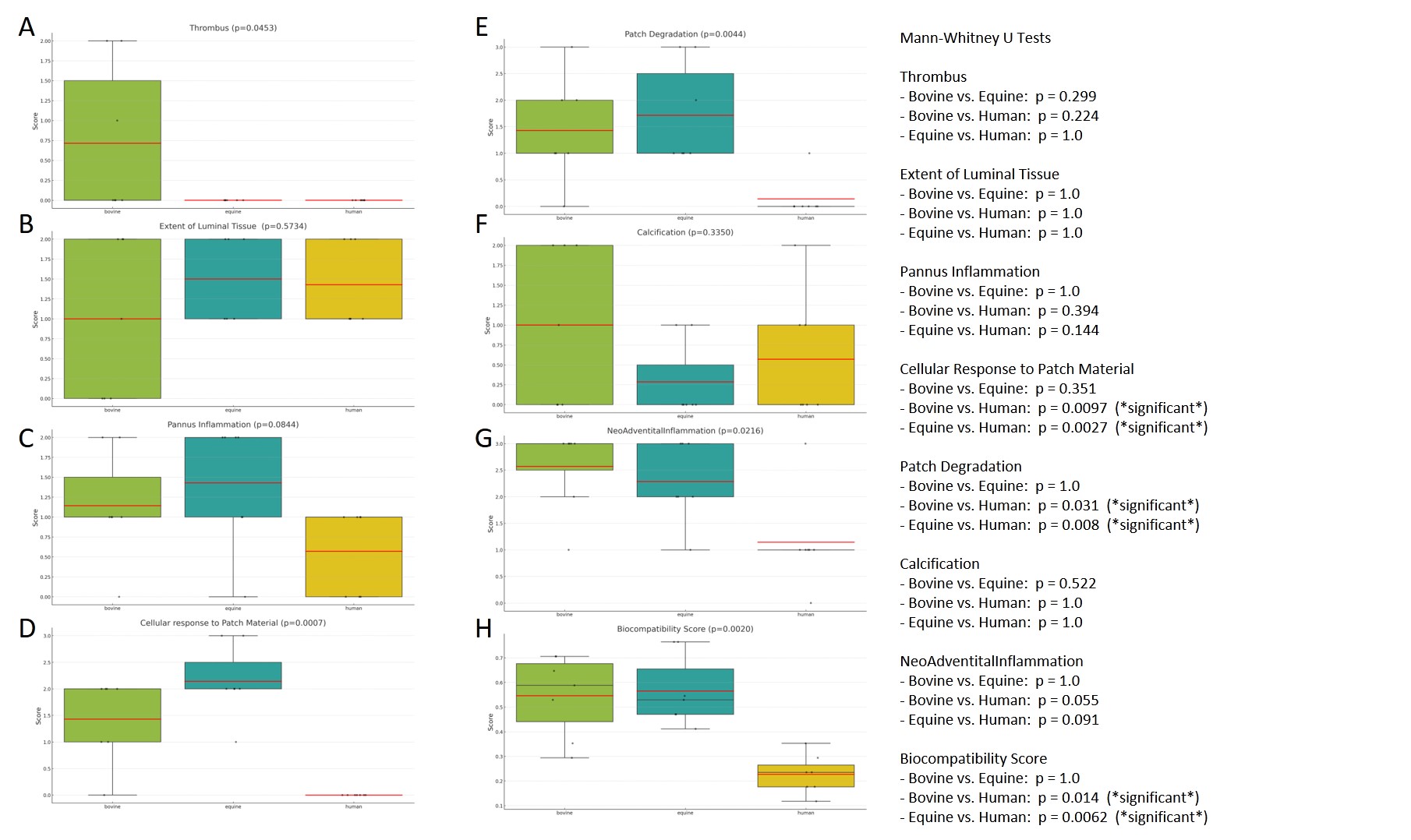

Supplement: ezae027_Supplementary_Data [file ezae027_supplementary_data.zip › Supplemental_Figure_1.jpg]

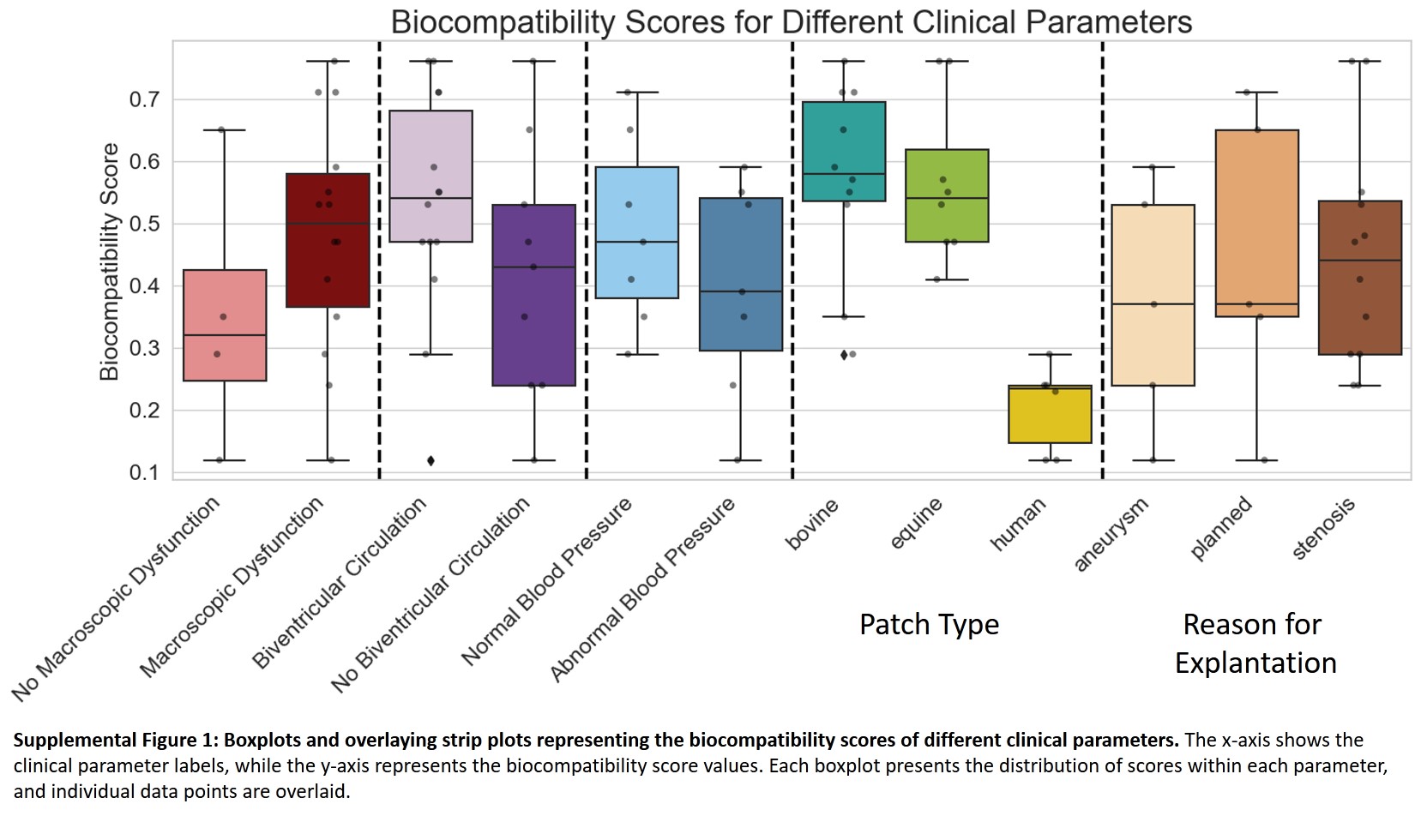

Supplement: ezae027_Supplementary_Data [file ezae027_supplementary_data.zip › Supplemental_Figure_2.jpg]
